# ﻿New species of *Asclepias* (Apocynaceae), *Baphia* (Leguminosae), *Cochlospermum* (Bixaceae) and *Endostemon* (Lamiaceae) from the Kalahari sands of Angola and NW Zambia, with one new combination in *Vangueria* (Rubiaceae)

**DOI:** 10.3897/phytokeys.232.110110

**Published:** 2023-09-20

**Authors:** David J. Goyder, Nina Davies, Manfred Finckh, Amândio Gomes, Francisco Maiato P. Gonçalves, Paulina Meller, Alan J. Paton

**Affiliations:** 1 Royal Botanic Gardens, Kew, TW9 3AE, London, UK National Geographic Okavango Wilderness Project, Wild Bird Trust Hogsback South Africa; 2 National Geographic Okavango Wilderness Project, Wild Bird Trust, Hogsback, South Africa Royal Botanic Gardens, Kew London United Kingdom; 3 Institute for Plant Science and Microbiology, University of Hamburg, Hamburg, Germany University of Hamburg Hamburg Germany; 4 Natural Science Faculty of Agostinho Neto University, Luanda, Angola Natural Science Faculty of Agostinho Neto University Luanda Angola; 5 Herbarium of Lubango, ISCED-Huíla, Lubango, Angola Herbarium of Lubango, ISCED-Huíla Lubango Angola

**Keywords:** *Ancylanthos*, *Casearia*, Cochlospermaceae, geoxyle, geoxylic suffrutices, Ilse von Nolde

## Abstract

Four new species are described from central and eastern Angola and adjacent NW Zambia. All occur in Kalahari sand savannas rich in endemic and more widely distributed geoxylic suffrutices. Despite being known from very few collections, the conservation status of one of these new species is assessed as Least Concern, as these grasslands are nutrient-poor, are in remote sparsely populated areas, and are not threatened with conversion to agriculture. The remaining three are treated as Data Deficient. In addition, one new combination is provided for *Ancylanthosrubiginosus* Desf. under *Vangueria* as *V.rubiginosa* (Desf.) Lantz is an illegitimate later homonym. We also make orthographic corrections to specific epithets commemorating Ilse von Nolde, a collector who made important collections from Quela in Malange in the 1930s.

## ﻿Introduction

Plant diversity in Angola is poorly documented with very uneven geographic coverage – much of the eastern half of the country and some northern provinces are largely devoid of georeferenced plant collections (Sosef et al. 2017; Goyder and Gonçalves 2019). Nevertheless, a Checklist of the plants of Angola was compiled by Figueiredo and Smith (2008), which serves as an invaluable baseline and point of reference for further studies. The indigenous flora of Angola is estimated to comprise 6850 species of vascular plant (Goyder and Gonçalves 2019).

Two initiatives have contributed data to the current publication. Firstly, a series of expeditions under the auspices of the National Geographic Okavango Wilderness Project (NGOWP) focussed initially on the headwaters of the Cuito and Cuanavale tributaries of the Okavango system in central Angola, then on river catchments further to the east in 2019. Most of these surveys were in Moxico Province, but neighbouring areas of Cuando Cubango with similar rainfall and vegetation were also documented. In parallel, The Future Okavango programme (TFO) of the University of Hamburg initiated a series of ecological studies in *miombo* and grassland sites around Chitembo, Bié Province, just to the west of the core NGOWP study area.

The NGOWP surveys culminated in a Checklist of the Cuito headwaters, documenting 417 species from the region (Goyder et al. 2018) with ten new country records for Angola, 108 new provincial records for Moxico, and nine species potentially new to science. Fieldwork in late 2019 in the Cuito headwater lakes area and further to the east resulted in additional localities for some of the undescribed species, two further undescribed species, and even more new country and provincial records. We here formally describe four of the new species – *Asclepiasminutissima* Goyder, *Baphiaarenicola* Goyder, F.M.P.Gonçalves & P.Meller, *Cochlospermumadjanyae* Goyder & Amândio Gomes and *Endostemonpalustris* A.J.Paton & Goyder. The new *Baphia* had also been encountered in several TFO field surveys. Additional novelties arising from NGOWP fieldwork in the region include two species of *Justicia* (Acanthaceae), *J.cubangensis* I.Darbysh. & Goyder and *J.moorei* I.Darbysh. & Goyder, described by Darbyshire and Goyder (2019), while *Barleria* sp. nov. of Goyder et al. (2018) has since been described as *B.thunbergiiflora* I.Darbysh. (Darbyshire et al. 2021) in a revision of *Barleria* in Angola and Namibia.

## ﻿Landforms, ecology and endemism of central/eastern Angola

Much of eastern Angola is overlain with deep deposits of Kalahari sand, which in the Cuito headwater zone are white, highly leached, and largely devoid of nutrients (Gröngröft et al. 2013a). The whole area seems to have been uplifted since the middle Miocene, 16 mya (Guillocheau et al. 2015). The plateau in NW Moxico is at an elevation of c. 1500–1600 m and is covered with extensive moist *miombo* woodland, a largely Zambesian vegetation type dominated by the genera *Brachystegia* Benth. and *Julbernardia* Pellegr. in Leguminosae subfam. Detarioideae. Some Congolian elements are also present in the region. Species composition is restricted compared with *miombo* woodlands on richer soils elsewhere in south tropical Africa (Goyder et al. 2018). Dense closed canopy stands of another detarioid legume, CryptosepalumexfoliatumDe Wild.subsp.pseudotaxus (Baker f.) P.A.Duvign. & Brenan, form patches of *miombo* forest within the more typical open canopy *miombo* woodland of the region. *Julbernardiapaniculata* (Benth.) Troupin commonly dominates the steep slopes of the river valleys, with *Brachystegiabakeriana* Hutch. & Burtt Davy generally the first woody *miombo* species to establish itself above the water table.

Tributaries of the Cuito River lie in steeply incised valleys around 150 m below the level of the surrounding plateau and their headwaters generally take the form of a wetland or seepage area surrounded by a narrow zone of exposed sand with a herbaceous or suffrutescent flora maintained free of woody vegetation by a combination of fire, frost and high water table (Maurin et al. 2014; Finckh et al. 2016; Goyder et al. 2018; Finckh et al. 2021).

Frequently, a little further downstream, a headwater lake occurs with deep open water and a fringing mat of peat, with its own suite of plants capable of surviving in this low pH environment. Wetlands are not generally known for high levels of plant diversity or endemism, although some species such as the insectivorous *Genliseaangolensis* R.D.Good appear to be restricted to peaty wetlands on Kalahari sand (Goyder 2016a). Clump or tussock-forming plants such as Eriocaulaceae and Xyridaceae are common, with several species of insectivorous Lentibulariaceae and Droseraceae also present. Cyperaceae are present but appear over-represented in pollen profiles from peat cores taken in the region (Lourenco et al. 2022). Indeed, the extent and significance of Angolan highland peat deposits has only been documented recently (Lourenco et al. 2022).

Further downstream, with the confluence of additional tributaries, the valleys generally become wider and the seasonally burned grassland zones more extensive. This is the habitat that is most significant botanically with many range-restricted and endemic species, including most of the species new to science described below. One notable valley grassland formation consists of extensive sand platforms perched above the level of the current watercourse; it is dominated by grasses and by geoxylic suffrutices, plants with considerable underground woody biomass and seasonal above-ground shoots. These lifeforms are adapted to above-ground disturbances such as fire and frost and were highlighted by White (1977) who, noting the prevalence of this lifeform in central and eastern Angola, referred to them as the underground forests of Africa. The below-ground biomass of these Kalahari sand grasslands rich in geoxylic suffrutices can approach the above-ground biomass of adjacent *miombo* woodlands (Gomes et al. 2021; Huntley 2023). Localities with significant extent of this habitat include the upper reaches of the Lungué-Bungo valley SE of Munhango and N of Chipola; confluences of headwater tributaries below both the Cuito and the Cuanavale River sources; and some of the Cusseque valley system near Chitembo, Bié Province, to the W of the Cuito headwater lake region, sampled extensively by the TFO programme. Three of the four species described here occur in this fire-adapted landscape. The fourth species, *Asclepiasminutissima*, was found at a slightly lower elevation in eastern Moxico Province, close to the Zambian border, at the edge of seasonally flooded Kalahari sand savannas on the western fringes of the Barotse floodplain.

Several globally rare species were noted in the high rainfall Kalahari sand savannas of Gabon’s Batéké Plateau (Walters et al. 2012; Walters et al. 2022) and our observations in Angola lead us to believe that the same is true here. An analysis of endemism in Kalahari sand floras will be the subject of a future contribution.

## ﻿Materials and methods

Morphological descriptions are based principally on examination of herbarium specimens at Kew, with smaller organs examined under a Leica MZ12.5 stereo-dissecting microscope fitted with a 10 mm graduated eyepiece graticule. Relevant type material not available for direct observation at K or BM was consulted through the JSTOR Global Plants portal www.plants.jstor.org, or the websites of individual herbaria such as COI www.uc.pt/en/herbario_digital or LISC https://actd.iict.pt/collection/actd:BIOHERB. Herbarium abbreviations follow Index Herbariorum (Thiers continually updated) except for the Collections Unit of Angola’s National Institute for Biodiversity and Protected Areas which is not listed by Thiers, and is here referred to as INBAC.

## ﻿Taxonomic treatment

### ﻿Apocynaceae: Asclepiadeae

#### ﻿ Asclepias minutissima

Taxon classificationPlantaeGentianalesApocynaceae

Goyder sp. nov.

625581C9-0685-5F0E-A94D-B897B76901E2

urn:lsid:ipni.org:names:77327146-1

##### Diagnosis.

*Asclepiasminutissima* appears most similar to *A.aurea* (Schltr.) Schltr. but differs in the campanulate rather than rotate to reflexed corolla, the ascending disposition of the corona rather than radiating from the column in *A.aurea*, the absence of the well-developed distal tongue to the corona of the latter species, and the shorter peduncles (1–3 cm rather than (3)5–14 cm in *A.aurea*).

##### Type.

Angola. Moxico Province: Mussuma plains, 50 km NE of Lumbala, Zambezi drainage, 13°45'49"S, 021°43'25"E, fl. 7 December 2019, *D.Goyder & F.Maiato* 9204 (holotype: K (K001334259); isotypes: INBAC, LUBA, PRE).

##### Description.

**Figure 1.**
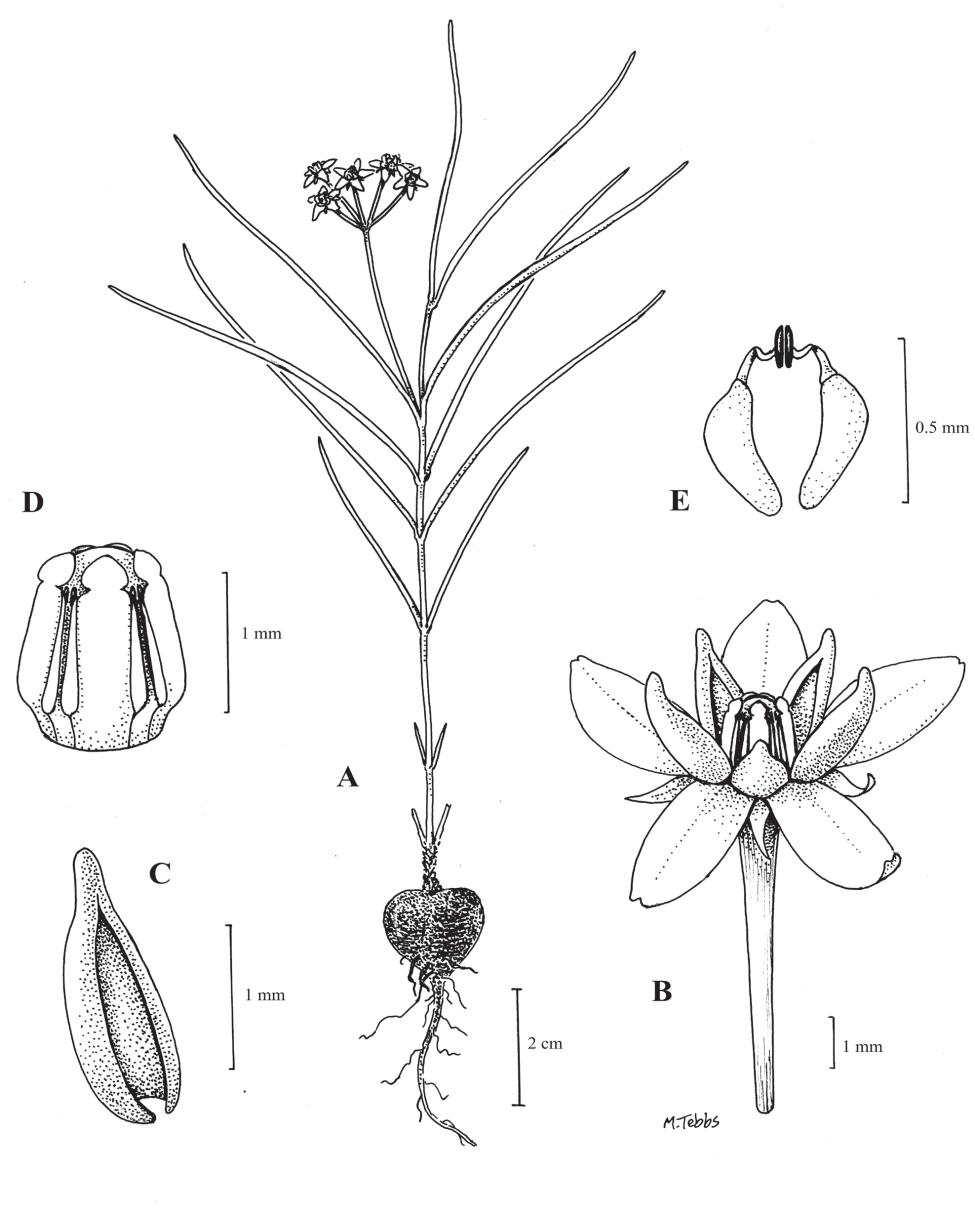
*Asclepiasminutissima***A** habit **B** flower **C** corona lobe **D** gynostegium **E** pollinarium. Drawn by Margaret Tebbs from *Richards* 17269.

Perennial herb with a single erect stem arising annually from a small napiform tuber, latex white; stems 8–15 cm long, minutely pubescent along two lines. Leaves sessile, 3–7 × 0.05 cm, filiform with inrolled margins, glabrous. Inflorescences terminal or extra-axillary, umbelliform, with 4–5 erect flowers; peduncles 1–3 cm long, minutely pubescent; pedicels c. 1 cm long, minutely pubescent. Sepals 1–1.5 mm long, narrowly to broadly triangular, glabrous. Corolla campanulate, lobes 3.5–4 × 1.5 mm, oblong, green or white, glabrous on both faces. Corona lobes 2–3 mm long, cucullate, lacking an apical tongue, pinkish cream or white. Anther wings 1 mm long. Stylar head flat. Follicles not seen. (Fig. 1).

##### Distribution and ecology.

**Map 1.**
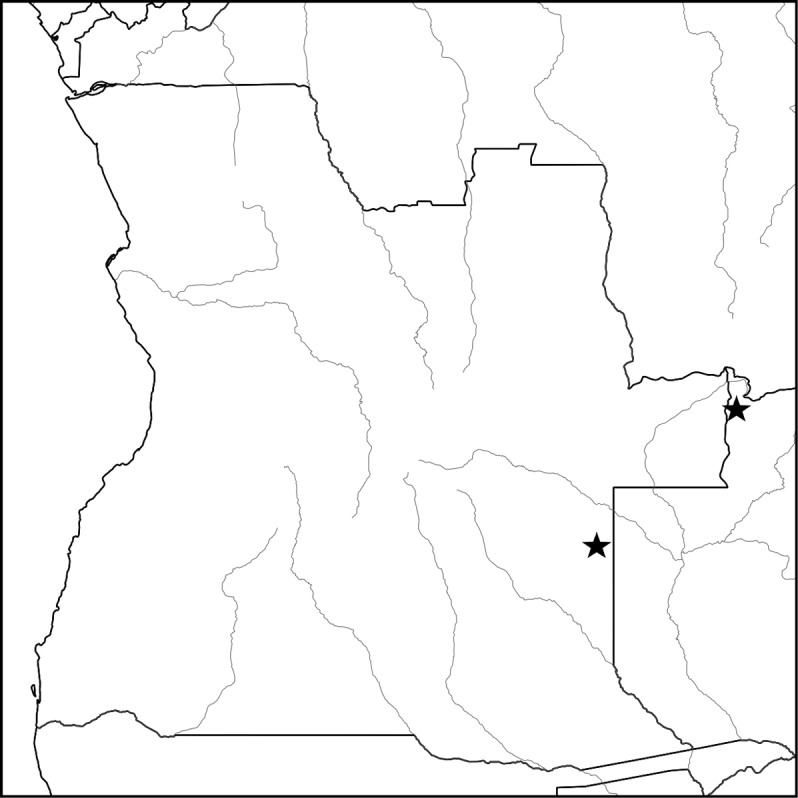
Known distribution of *Asclepiasminutissima* (eastern Angola and NW Zambia).

Known from a single collection in eastern Angola and one in NW Zambia. The Angolan population consisted of several scattered individuals on a broad open sandy plain just above the water table. Both the Angolan and the Zambian localities are on Kalahari sand deposits, and the Angolan collection was associated with common geoxylic suffrutices of the region such as *Parinaricapensis* Harv., Syzygiumguineense(Willd.)DC.subsp.huillense (Hiern) F.White, *Eugeniamalangensis* (O.Hoffm.) Nied., LanneagossweileriExell & Mendonçasubsp.gossweileri and Cryptosepalumsp. aff.mimosoides Welw. ex Oliv. Altitude 1100–1300 m. (Map 1).

##### Conservation status.

*Asclepiasminutissima* is known from two localities some 350 km apart, but is inconspicuous and easily overlooked and is likely to be more common than the herbarium records suggest. Both localities are in nutrient-deficient sandy environments unsuitable for agriculture, and with little threat of habitat transformation as human settlements are few and far between. The new species is therefore provisionally assessed as Data Deficient.

##### Additional specimens examined.

Zambia. Mwinilunga District, 16 km along road from Matonchi Farm, 11°39'S, 24°03'E, fl. 17 November 1962, *Richards* 17269 (K).

##### Taxonomic notes.

Only three species of *Asclepias* were reported from Angola by Goyder (2008). *Asclepiasbaumii* Schltr., known only from the type collection which was destroyed in Berlin, is almost certainly synonymous with *A.aurea*, as is *A.radiata* S.Moore (Goyder 2009). *Asclepiasrandii* S.Moore is also present. Following the transfer of *Odontostelma* Rendle to *Asclepias* by Goyder (2009), *A.minor* (S.Moore) Goyder, also occurs in the country. All of these species occur in scattered populations with few individuals, as is common for *Asclepias* and allied genera in tropical Africa. As a result, they tend to be collected very infrequently and few herbarium records exist for any of them in Angola.

*Asclepiasrandii* is a much more robust plant than the other species and has pubescent stems and leaves. *A.aurea* and *A.minor* are glabrous and are slender herbs. *Asclepiasminor* has a corona which is much reduced, not even reaching the base of the anther wings and has a short ventral appendage. So the species most similar to our new collection appears to be the highly variable *A.aurea*, which occurs across Namibia, southern Angola, Zambia, the Katanga region of the D.R.Congo, Zimbabwe, northern provinces of South Africa, Eswatini (Swaziland) and Lesotho (Goyder 2009).

*Asclepiasaurea* has rotate to reflexed corolla lobes, corona lobes which radiate from the column and are extended into a long distal tongue, and longer peduncles. In addition to the rather subtle morphological characters that distinguish the new species from *A.aurea*, its ecological requirements, close to the water table on leached Kalahari sand, are probably also significant. *Asclepiasaurea* occurs on richer soils. The new taxon was mentioned by Goyder et al. (2020: 276) in a note under the related *A.aurea* but the Angolan (type) collection was cited incorrectly as *Goyder & Gonçalves* 4809.

### ﻿Bixaceae: Cochlospermeae

#### ﻿ Cochlospermum adjanyae

Taxon classificationPlantaeMalvalesCochlospermaceae

Goyder & Amândio Gomes sp. nov.

02230A6D-3A07-5E0D-A7B0-FA1F8D8F9C12

urn:lsid:ipni.org:names:77327147-1

##### Diagnosis.

*Cochlospermumadjanyae* differs from all African species of the genus in possessing palmatisect rather than palmatifid or lobed leaves, with discrete leaflets rather than partially connate lobes.

##### Type.

Angola. Moxico Province: Lungué-Bungo valley, 50 km S of Munhango, near Lungué-Bungo bridge, 12°36'50"S, 018°47'59"E, fl. 20 November 2019, *D.Goyder & A.Gomes* 9002 (holotype: K (K001334241); isotypes: INBAC, LUBA, PRE).

##### Description.

**Figure 2.**
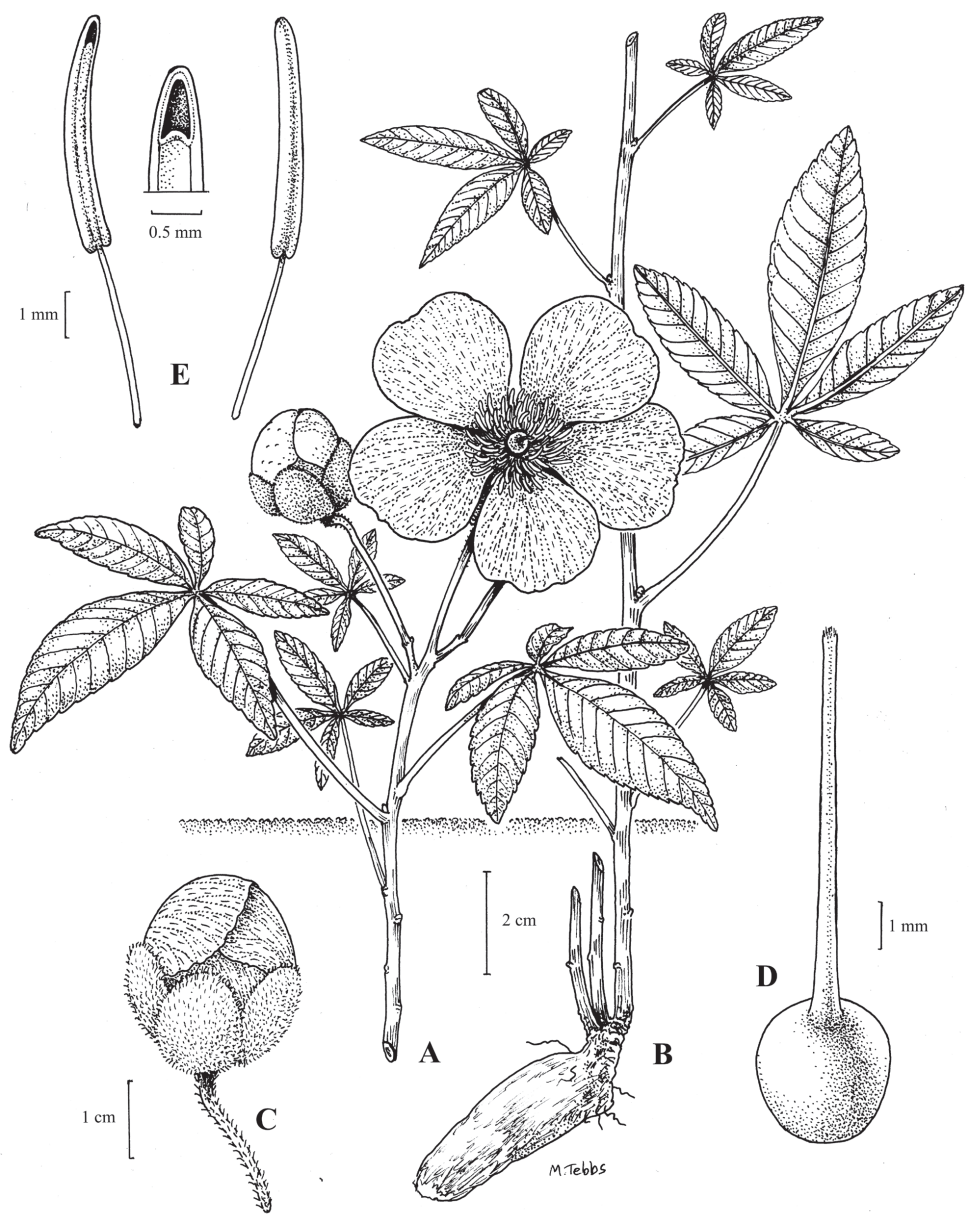
*Cochlospermumadjanyae***A, B** habit **C** flower **D** ovary and style **E** stamens. Drawn by Margaret Tebbs from *Goyder & Gomes* 9002.

**Figure 3.**
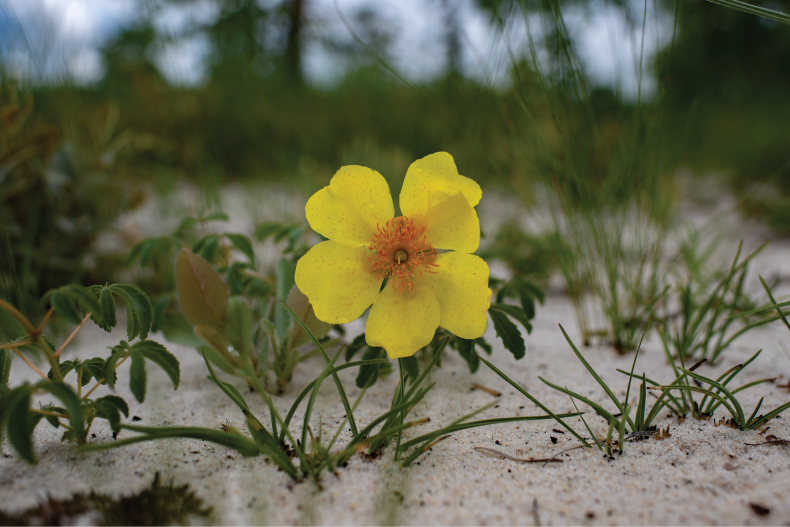
*Cochlospermumadjanyae*. Photographed by Chris Boyes at the type locality.

Geoxylic suffrutex forming diffuse but discrete patches several metres across; above-ground stems 10–20 cm tall, sub-erect, glabrous, burned off in the dry season. Leaves palmate with (4–)5 leaflets; stipules c. 3 mm long, narrowly triangular; petioles (2–)4–6 cm long, glabrous except for a minute rusty pubescence at the junction with the leaflets; leaflets reducing in size from the central leaflet to the lateral and basal ones, central leaflet 3–5 cm long, 1.2–2 cm wide, elliptic to slightly obovate, acute or occasionally obtuse apically, the base somewhat cuneate, margins serrate at least in the upper half, glabrous except for a minute rusty pubescence at the junction with the petiole adaxially. Inflorescences minutely rusty-puberulent, terminal on the leafy shoots, with 1–3 flowers; peduncles 2.5–3 cm long; sepals subequal, 11–15 × 7–8 mm, broadly ovate or elliptic, rounded apically, minutely rusty-puberulent and with occasional dark streaks, the outer pair more deeply coloured than the inner three; petals c. 3 × 2–2.3 cm, obovate, rounded or slightly emarginate, bright yellow with linear red streaks. Stamens numerous (80+), yellow; anthers c. 5 mm long, straight or weakly curved, apical pore 0.5–1 mm long. Ovary c. 2 mm in diameter, glabrous. Fruit not seen. (Figs 2, 3).

##### Distribution and ecology.

**Map 2.**
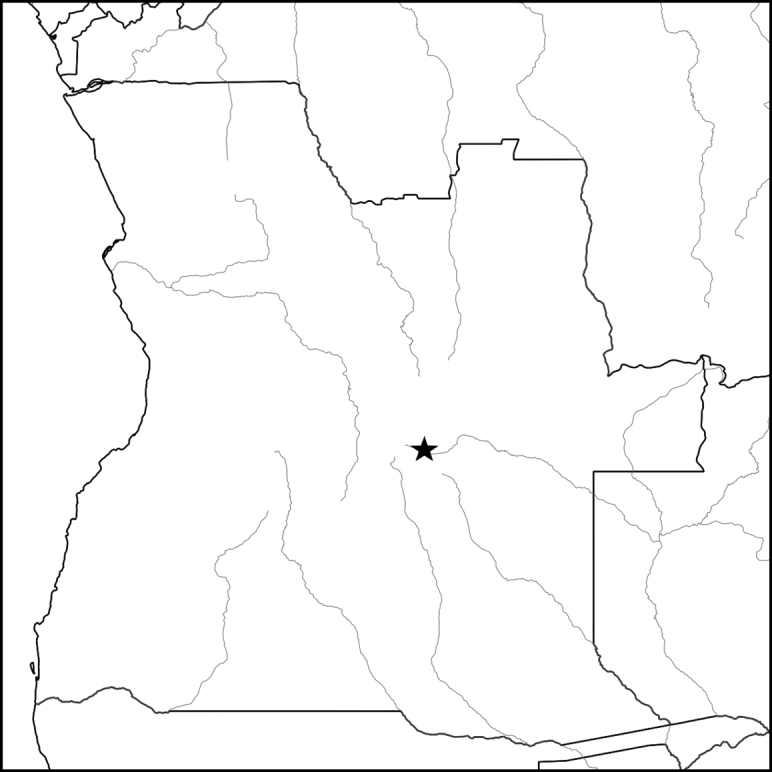
Known distribution of *Cochlospermumadjanyae*.

Found only once in flower in grassland rich in geoxylic suffrutices on deep Kalahari sand. Material in bud had been encountered, but not collected, the day before in a similar grassland some 15 km to the NW along with the new species of *Baphia* described below and other geoxyles such as *Sclerocrotonoblongifolius* (Müll.Arg.) Kruijt & Roebers, *Parinaricapensis*, *Entadaarenaria* Schinz and *Englerophytummagalismontanum* (Sond.) T.D.Penn. The fact that this conspicuously flowered species was seen only on the November 2019 expedition and not on earlier ones through the same valley system (late rainy season; early and mid-dry season) suggests that populations are highly localised and that the flowering period is short, at the end of the dry season, and perhaps dependent on rainfall following fire. Altitude 1285–1340 m. (Map 2).

##### Etymology.

The specific epithet honours Adjany Costa who was part of the core National Geographic Okavango Wilderness Project headwaters team from the first expedition in 2015 until she left to pursue academic studies at Oxford University in 2019. Initially, she supported freshwater fish specialists Paul Skelton and Ben van der Waal, recording and preserving freshwater fish diversity from the Cuito source to the Delta, before developing the communities programme with Chris and Steve Boyes. She was a National Geographic Young Explorer, starred in the National Geographic documentary film “Into the Okavango”, and was awarded the United Nation’s Young Champions of the Earth Prize for Africa in 2019.

##### Conservation status.

While *Cochlostemumadjanyae* is known from a single locality, these geoxyle-rich grasslands are not currently threatened as this nutrient-poor sandy environment is not conducive to agricultural development. The environment does not support many human settlements, which are few and far between. The species is probably best assessed as Data Deficient.

##### Taxonomic notes.

*Cochlospermum* Kunth is a pantropical genus of around 16 species of trees, shrubs and geoxylic suffrutices, or if expanded to include the herbaceous neotropical genus *Amoreuxia* DC., 20 species. *C.noldeae* Poppend. from NE Angola, *C.macnamarae* Hislop, K.R.Thiele & Brassington and *C.arafuricum* Cowie & R.A.Kerrigan from Australia (Poppendieck 2004 (see nomenclatural note below), Hislop et al. 2013, Cowie and Kerrigan 2015) were described after Poppendieck’s (1980) monograph of the group where he had recognised 12 species. Irrespective of the circumscription of the genus, molecular evidence presented by Johnson-Fulton and Watson (2017) supports the monophyly of CochlospermumsubgenusCochlospermum sensu Poppendieck (1980), which comprises two species from Central and South America (*C.vitifolium* (Willd.) Spreng. and *C.regium* (Mart. ex Schrank) Pilg.), and all the paleotropical species. Johnson-Fulton and Watson (2017) argue that the geoxylic habit evolved just once from an ancestral arboreal lifeform – all but one of the African taxa are geoxyles, together with a single neotropical species, *C.regium*, from *cerrado* (savanna) regions of Brazil, Paraguay and Bolivia. The remaining species are trees or shrubs.

Species of Cochlospermumsubg.Cochlospermum have a single apical pore to the anthers, and in addition to growth form, can be distinguished by leaf indentation or lobing, indumentum, and the position and timing of flowers on the shoots. A collection made in the geoxyle-rich Lungué-Bungo valley grasslands of Moxico in November 2019 is unique in the African species in having palmatisect leaves, divided to the base rather than being merely lobed. The only other taxon in the subgenus with this leaf character is the nomenclaturally illegitimate C.gillivrayiBenth.subsp.gregorii (F.Muell.) Poppend., an Australian tree. The Angolan material is described as a new species. It is perhaps closest morphologically to *C.wittei* Robyns from the Upemba region of Katanga. *Cochlospermumwittei* is also associated with savanna and woodland on sand plateaux (Poppendieck 1980) but these are nearly 1000 km to the NE of the Lungué-Bungo grasslands.

### ﻿Key to African species of *Cochlospermum*

**Table:** 

1	Trees or shrubs at least 4 m tall	***C.angolense* Welw. ex Oliv.**
–	Geoxylic suffrutices or low shrubs with annually produced shoots less than 4 m tall	**2**
2	Flowering mostly near ground level after fires and before the development of leafy shoots; savanna regions north of the equator	***C.tinctorium* A.Rich.**
–	Flowering towards the tip of leafy shoots	**3**
3	Leaves 3-lobed, the apex of the lobes attenuate; anthers 7 mm long; NE Angola: Malange	***C.noldeae* Poppend.** ^*^
–	Leaves mostly 5–7-lobed, the apices acute, obtuse or rounded; anthers 4–6 mm long	**4**
4	Leaves palmately compound with leaflets free to the base; eastern Angola: Moxico	***C.adjanyae***
–	Leaves palmately lobed, the lobes connate for at least some of their length	**5**
5	Leaves with lobes connate for at least half their length, lobes rounded or obtuse apically; leaves generally silvery-white beneath; West African savannas	***C.planchonii* Hook.f. ex Planch.**
–	Leaves with lobes connate for less than half their length, lobes obtuse or acute apically; leaf indumentum variable	**6**
6	Leaves with lobes connate for ¼–½ of their length; Central African Republic	***C.intermedium* Mildbr.**
–	Leaves with lobes connate for less than ¼ of their length; DR Congo: Katanga	**7**
7	Leaves glabrous beneath; sepals minutely puberulous; ‘forest’ understorey	**C.witteisubsp.wittei**
–	Leaves silvery-white beneath; sepals minutely tomentose, greyish; savanna on Kalahari sand	**C.witteisubsp.incanum (Robyns) Poppend.**

Ilse von Nolde, who lived in Quela, eastern Malange Province where she and her husband farmed coffee, made important collections of plants from the region between 1928 and 1938 (Esdorn 1972; Poppendieck 2004; Figueiredo et al. 2008; Figueiredo et al. 2020). While the top set of her collections in Berlin appears to have been destroyed, some material is duplicated at BM, COI, LISC and MO, and her botanical notes and illustrations are preserved at HBG (Poppendieck 2004). Several species were named after her and while most have the correct termination two, *Cochlospermumnoldei* Poppend. and *Casearianoldei* A.Fern. & Diniz, end in -i rather than -ae. -i would be the correct termination for a male collector. We correct these epithets here to *Cochlospermumnoldeae* and *Casearianoldeae*, in accordance with Art. 60.8a of the International Code for Nomenclature (Turland et al. 2018).

### ﻿Lamiaceae: Ocimeae

#### ﻿ Endostemon palustris

Taxon classificationPlantaeLamialesLamiaceae

A.J.Paton & Goyder sp. nov.

B3859CC8-5EBA-5390-8D5A-B828A10A4B47

urn:lsid:ipni.org:names:77327148-1

##### Diagnosis.

Differs from *E.tubulascens* (Briq.) M.Ashby in the little-branched habit, the sessile rather than petiolate and linear rather than elliptic leaves, the fewer-flowered (4–6-flowered rather than 6–10-flowered) verticils in the inflorescence, the pale violet rather than pinkish white flowers and the longer calyx (fruiting calyx 8–9 mm long rather than 5.5–6 mm).

##### Type.

Angola. Moxico Province: Confluence of Cuito River and its 1^st^ tributary, the Kalua River, c. 65 km SSW of Munhango, 12°44'55"S, 018°21'16"E, fl. 20 Oct. 2016, *D.Goyder & F.Maiato* 8762 (holotype: K (K001333409); isotypes: INBAC, LUBA).

##### Description.

**Figure 4.**
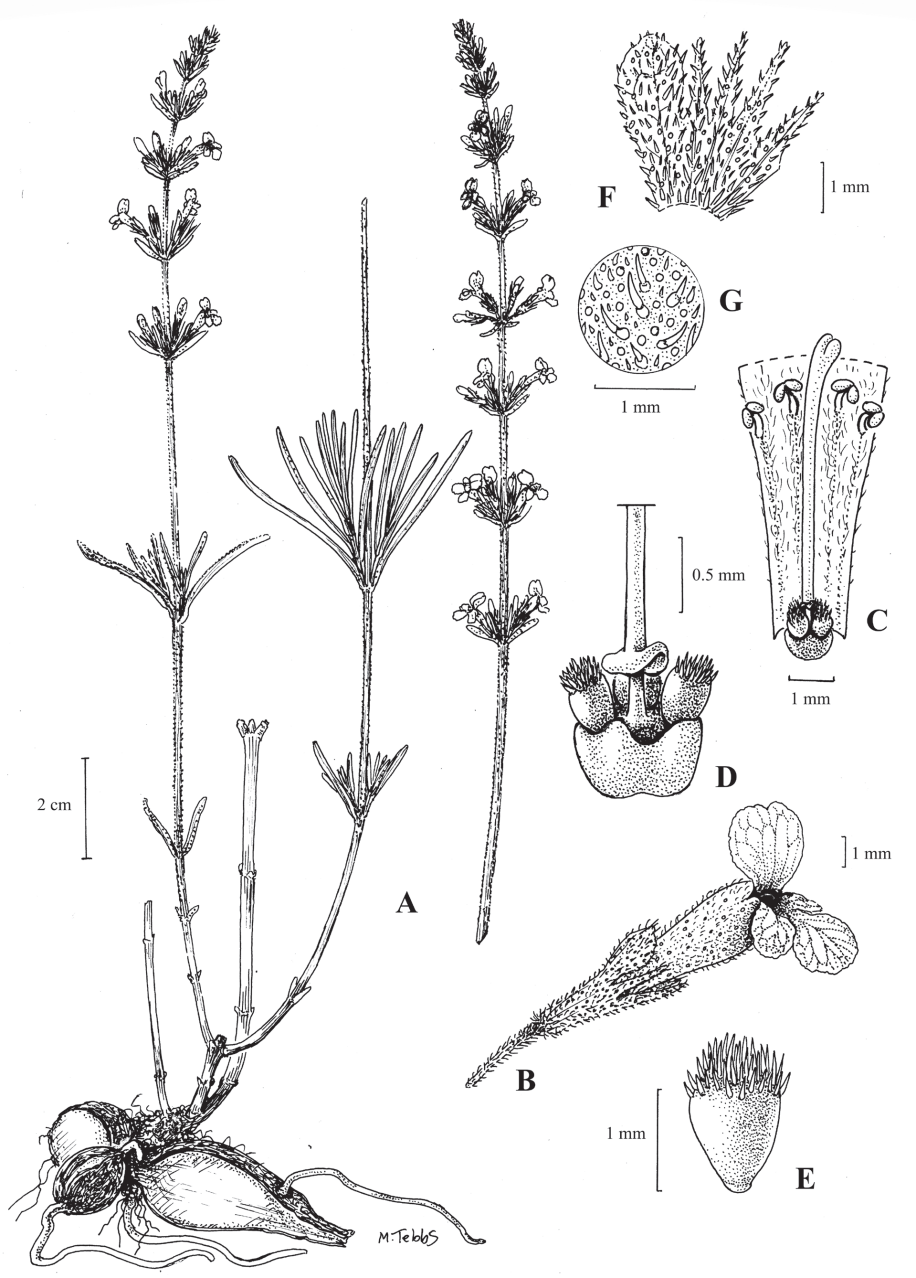
*Endostemonpalustris***A** habit **B** flower **C** opened corolla showing androecium and gynoecium **D** base of gynoecium showing three of the four nutlets and the gynobasic style **E** individual nutlet **F** calyx **G** indumentum. Drawn by Margaret Tebbs from *Goyder & Maiato* 8762.

Aromatic perennial suffrutex with few stems arising from a thick woody rootstock; stems erect, 15–30 cm tall, branched near the base, square in section above, more rounded below, pubescent with both glandular and eglandular hairs. Leaves verticillate, ascending, sessile, linear and folded along the midvein, 2.5–5 cm long, 0.1–0.2 cm wide, pubescent. Inflorescence lax with 4–6-flowered verticils 1.5–2.5 cm apart; bracts lanceolate or narrowly ovate, 3.5–4.5 mm long; pedicels 1–5 mm long, longer in lower verticils. Calyx 5 mm long at anthesis, pubescent with spreading hairs and sessile glands, posterior lip purplish; fruiting calyx 8–9 mm long. Corolla pale violet, 8–10 mm long; tube 6–7 mm long, straight, parallel-sided, dilating at the throat. Filaments c. 0.5 mm long, glabrous or pilose. Ovaries pubescent at apex. (Fig. 4).

##### Distribution and ecology.

**Map 3.**
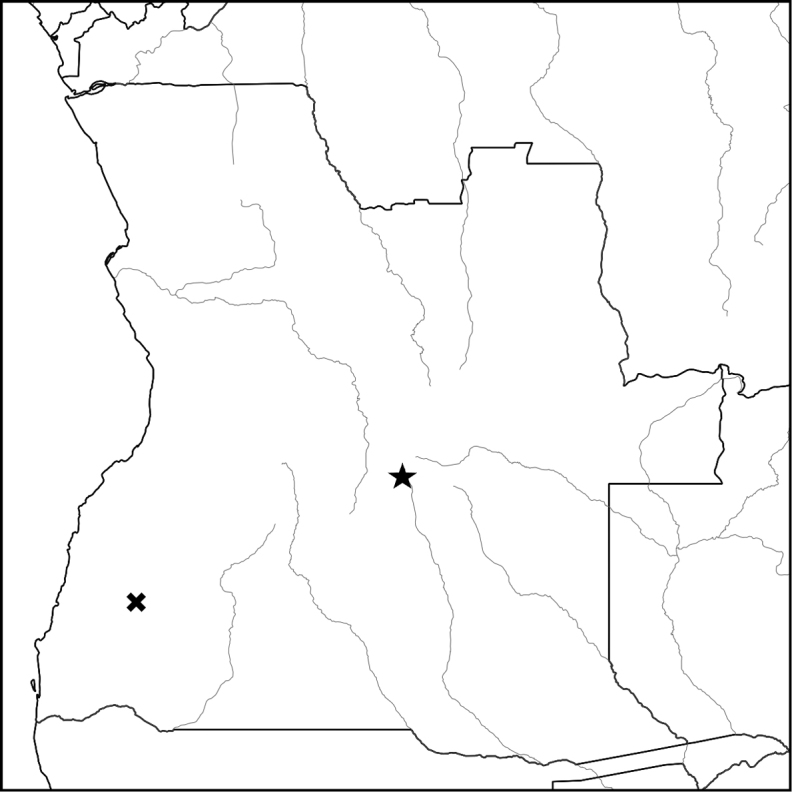
Known distribution of *Endostemonpalustris* (star), and of the related species *E.tubulascens* (cross).

Known only from the type collection close to the source of the Cuito River. It was found towards the upper edge of a marsh fringing the river. (Map 3).

##### Conservation status.

Although known from a single locality, *Endostemonpalustris* occupies a habitat that is extensive within the upper catchment of the Cuito and its tributaries. The area is not threatened with agricultural development being both nutrient poor and many kilometres from any human habitation, but with so little information the species is provisionally assessed as Data Deficient.

##### Taxonomic notes.

*Endostemon* N.E.Br. is an isolated genus of 20 species within the tribe Ocimeae, with two centres of endemism – Angola and the Horn of Africa. It can be recognised within the *Orthosiphon* Benth. group of genera by its short, villous staminal filaments, an expanded shield-like base to the style, and pollen with alternating wide and narrow mesocolpia (Paton et al. 1994; Ryding et al. 2003; Paton et al. 2004). With the exception of *E.tereticaulis* (Poir.) M. Ashby which is in Endostemonsect.Oblongi Ayob. ex A.J.Paton, Harley & M.M.Harley, all of the Angolan species fall within Endostemonsect.Endostemon. This section is characterised by a relatively short calyx tube with an open glabrous throat, and the form of the lateral calyx lobes – the posterior margins of these lobes are not extended towards the upper lip of the calyx. *Endostemontubulascens*, to which this new species is compared, appears largely restricted to the high escarpment zone around Lubango, Huíla Province, Angola.

### ﻿Leguminosae: Papilionoideae

#### ﻿ Baphia arenicola

Taxon classificationPlantaeFabalesFabaceae

Goyder, F.M.P.Gonçalves & P.Meller sp. nov.

1B3E6B92-C5C0-554B-B951-42D7A268AA2F

urn:lsid:ipni.org:names:77327149-1

##### Diagnosis.

Most similar morphologically to *B.massaiensis* Taub., from which it can be readily distinguished by its geoxylic lifeform, flowering and fruiting at ground level on short, prostrate above-ground shoots, and the villous suture of the keel petal.

##### Type.

Angola. Moxico Province: tributary of the Lungué-Bungo River 42 km SSE of Munhango, 12°31'34"S, 018°40'13"E, fl. 22 October 2016, *D.Goyder & F.Maiato* 8772 (holotype: K (K001333933); isotypes: INBAC, LUBA).

##### Description.

**Figure 5.**
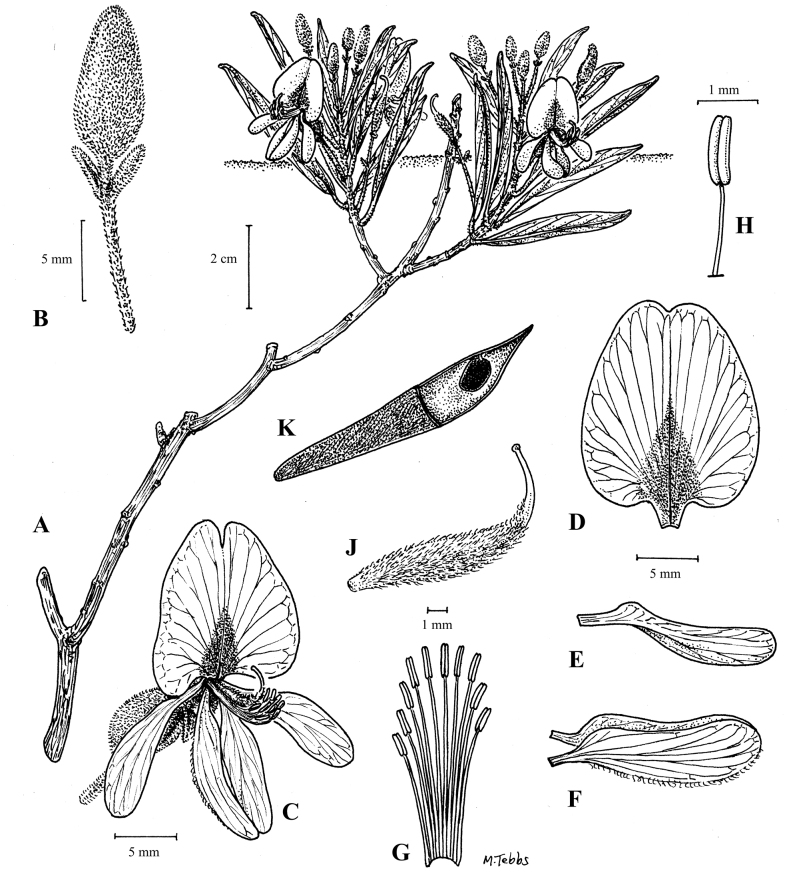
*Baphiaarenicola***A** habit **B** bud **C** open flower **D** standard petal **E** wing petal **F** keel petal **G** androecium **H** stamen **J** gynoecium **K** fruit. Drawn by Margaret Tebbs from *Goyder & Maiato* 8772 (flowering material) and *Gomes & Maiato* 161 (fruit).

**Figure 6.**
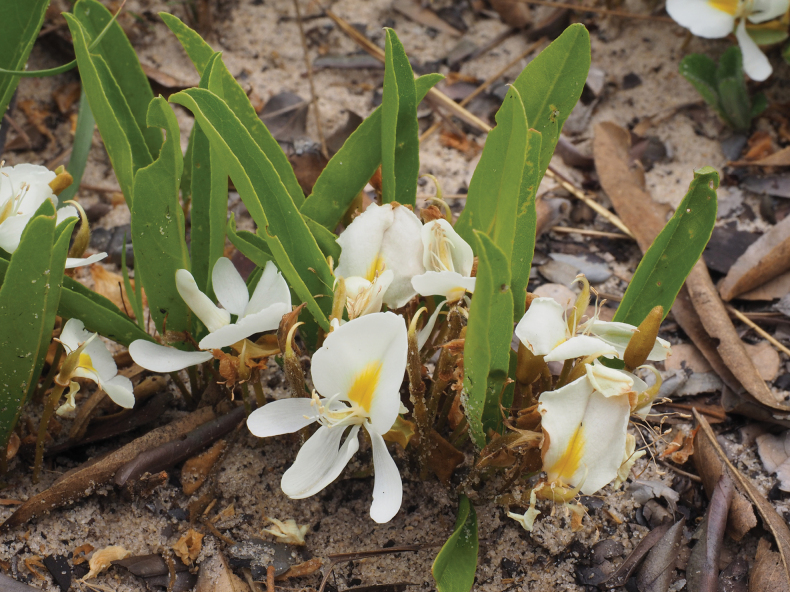
*Baphiaarenicola*. Photographed by David Goyder at the type locality.

Geoxylic suffrutex forming large patches; above-ground shoots prostrate, 5–10 cm long, pubescent, arising from extensive woody below-ground stems. Leaves unifoliolate; stipules linear, 3–4 mm long, densely pubescent with silvery hairs; petiole 3–5 mm long, pulvinus barely apparent; leaflet narrowly obovate-oblong and generally folded along the midvein, 4–6 cm long, 1–2 cm wide, obtuse apically, tapering somewhat towards the base, ± glabrous except for the major veins beneath. Inflorescences with an indumentum of greyish or golden hairs; flowers 1–3 in sessile or subsessile axillary fascicles; pedicels 15–20 mm long; bracteoles 1–2 mm below the calyx, caducous. Calyx 7–10 mm long, spathaceous. Petals white, the standard with a yellow triangular mark towards the base; standard 10–12 × 8–10 mm, emarginate; wings c. 10 × 2 mm; keel 10–12 × 2.5–3 mm, lower suture villous distally. Stamens 10, free. Ovary 6–9 mm long, villous, with a glabrous upturned style. Legume c. 7 cm long, c. 1.5 cm wide, brown. Seeds c. 10 mm long, dark brown or black. (Figs 5, 6).

##### Distribution and ecology.

**Map 4.**
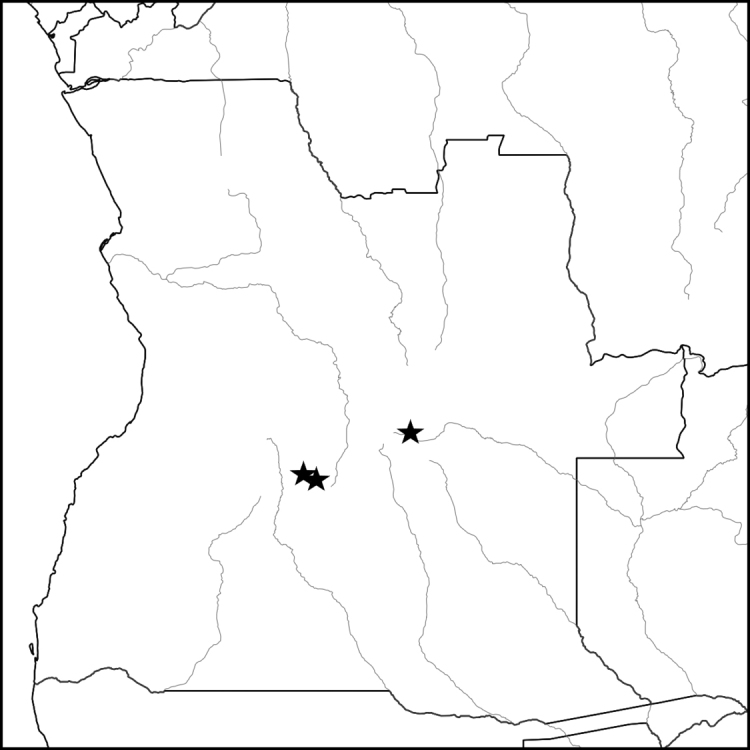
Known distribution of *Baphiaarenicola* (central Angola).

Flowering in October and November in the late dry season or at the onset of the rains; fruiting in February. Known from three sites, the first in the Lungué-Bungo river system of western Moxico Province at an elevation of around 1350 m, the second in the Cusseque River system of Bié Province some 200 km to the SW of the Moxico site at around 1530 m, and the third just over the watershed into the Cacuchi River valley which drains into the Rio Cuchi and is around 1540 m. The three sites are similar topographically, with broad fossil river terraces and sandy alluvial deposits rich in geoxylic suffrutices (Gröngröft et al. 2013b, Revermann et al. 2013, Goyder et al. 2018). (Map 4).

##### Conservation status.

*Baphiaarenicola* is known from three localities, two of which have vouchered herbarium collections. TFO project made many unvouchered observations of the plant in the Cusseque and Cacuchi River valleys and it is clear that there are extensive populations of this species. There are no significant threats to these nutrient-poor grasslands rich in geoxylic suffrutices as the environment is not conducive to agricultural development. *B.arenicola* is therefore provisionally assessed as Least Concern.

##### Additional specimens examined.

Angola. Bié Province: Cusseque, TFO core site relevé 23324, 13°41'53"S, 017°06'43"E, fl. 29 October 2011, *Finckh* 132753 (HBG, K); Cusseque, TFO core site relevé 23349, 13°41'53"S, 017°07'48"E, fl. 2 November 2011, *Revermann* 132895 (HBG, K); Cusseque village, Chitembo, 13°43'21"S, 017°05'53"E, fr. 17 February 2014, *Gomes & Maiato* 161 (LUBA); Cusseque 15 September 2019, *Finckh* 145383A (HBG, LUBA); Cusseque, 28 January 2020, *Finckh* 145352B (HBG, LUBA).

##### Taxonomic notes.

*Baphia* Afzel. ex G.Lodd is a genus of around 50 species of woody legumes which has diversified across tropical Africa (Soladoye 1985, Goncharov et al. 2011). A single species extends its distribution to the white sands of NW Madagascar, with a second species endemic to that region (Stirton and Du Puy 1992). Members of the genus are recognised readily by their unifoliolate leaves, free rather than united stamens, and flat, dark brown seeds lacking an aril. Seeds of other genera of the unifoliolate Baphioid clade of Lewis et al. (2005) are globose, arillate, and bicoloured or otherwise brightly coloured (Cheek et al. 2014). *Baphia* species occur in evergreen forest, thicket and woodland with several species restricted to white sands (Stirton and Du Puy 1992, Brummitt 2007, Mackinder and Clark 2012, Cheek et al. 2014). While most species are shrubs or small trees, *B.aurivellerea* Taub. from NE Angola and western DR Congo can be suffrutescent, but this species still possesses erect woody shoots 20 cm to two metres above ground level. In contrast, an undescribed species encountered in central Angola flowers at ground level on short, prostrate above-ground shoots and is here formally described as *Baphiaarenicola*.

Most floral characters invite comparison with the locally common woodland species *B.massaiensis*, with its spathaceous calyx split longitudinally down a single line, bracteoles longer than wide and positioned shortly below the apex of the pedicel, glabrous staminal filaments, and pubescent ovary. The keel petal which is somewhat villous along its line of fusion, however, suggests links to *B.bequaertii* De Wild., another *miombo* woodland species of the region. Preliminary molecular analyses by one of us (PM) places the new taxon close to the latter species, with estimated divergence times from *B.bequaertii* less than 1 mya. Divergence times from *B.massaiensis*, on the other hand, are estimated to be between 11 and 27 mya.

### ﻿Rubiaceae: Vanguerieae

#### ﻿ Vangueria fulgida

Taxon classificationPlantaeGentianalesRubiaceae

(Welw. ex Hiern) Goyder & N.M.J.Davies comb. nov.

58D7735C-BB2A-543D-B178-F99DAEC4B412

urn:lsid:ipni.org:names:77327150-1

Ancylanthos fulgidus Welw. ex Hiern in Oliv. (ed.), Fl. Trop. Afr. 3: 159 (1877). Type: Angola, Huíla, Mumpulla to Lopollo, Oct. 1859, *Welwitsch* 3160 (lectotype: LISU (LISU208624) designated here; paralectotypes: BM, K, LISU, P, PRE). (Basionym).
Ancylanthos rubiginosus Desf., Mém. Mus. Hist. Nat. 4: 5 (1818). Vangueriarubiginosa (Desf.) Lantz, Pl. Syst. Evol. 253: 181 (2005), non V.rubiginosa K.Schum., Bot. Jahrb. Syst. 23: 457 (1897) [= Rytigyniarubiginosa (K.Schum.) Robyns, Bull. Jard. Bot. État Bruxelles 11: 209 (1928)]. Type: Angola [without locality or collector, but probably Benguela/Huila Plateau, 1785–1787, *J.J. da Silva* (Exell & Mendonça 1956: IX & XI, following note on specimen at P)] (holotype: P (P00138559)).
Ancylanthos ferrugineus Welw., J. Trav. Nat. Hist. 1: 29 (1868), nomen nudum.
Ancylanthos bainesii Hiern in Oliv. (ed.), Fl. Trop. Afr. 3: 160 (1877). Type: Baines s.n. (lectotype: K (K000412071) designated by Robyns 1928: 329).

##### Taxonomic and nomenclatural notes.

*Vangueriafulgida* is a small woody species with conspicuous orange flowers. It can form single-stemmed plants to 1.5 m in height, but over much of its range it behaves as a geoxylic suffrutex, forming patches of much shorter above-ground shoots that are burned off each year. It is found mostly on Kalahari sands and is widely distributed across Angola, western Zambia, Botswana, Namibia and Zimbabwe (Bridson 1998; Figueiredo 2008a, b).

Molecular studies of the tribe Vanguerieae A.Rich. ex Dumort. (Lantz and Bremer 2004; Lantz and Bremer 2005; Razafimandimbison et al. 2009) have shown that the species of *Ancylanthos* Desf. are showy members of *Vangueria* Juss., and combinations under the latter were made in Lantz and Bremer (2005). However, *Vangueriarubiginosa* (Desf.) Lantz is a later homonym of *V.rubiginosa* K.Schum. (Schumann 1897: 457) and is therefore illegitimate.

The earliest synonym listed by Bridson (1998), *Ancylanthosferrugineus* Welw. (Welwitsch 1868a: 29), must be treated as a *nomen nudum* because “very pretty” does not constitute a validating description under the International Code of Nomenclature for algae, fungi, and plants (Turland et al. 2018). No specimen was cited but, as Welwitsch was writing about the Pedras Negras of Pungo Andongo, Angola it can be inferred that *Welwitsch* 3159 from this region is probably this plant. Welwitsch’s second account of the Pedras Negras (Welwitsch 1868b), makes no mention of the plant.

*Ancylanthosbainesii*Hiern (1877) and *A.fulgidus* Welw. ex Hiern (1877) were published simultaneously and the epithets are both available under *Vangueria*, so either could be used as a basionym for a new combination to replace the illegitimate *V.rubiginosa* (Desf.) Lantz – we have chosen to use *A.fulgidus* described from the Huíla Plateau of Angola, a name more widely adopted than *A.bainesii* (Hiern 1898; Schumann 1903: 390; Durand and Durand 1909: 271; De Wildeman 1910: 424, 1913: 153, 1925: 204; Robyns 1928). We designate the LISU specimen bearing Welwitsch’s handwritten note as the lectotype of this name.

Hiern (1877) cited two specimens in his description of *Ancylanthosbainesii*. One, without a locality, was just cited as “T.Baines!”. The second is the Chapman & Baines collection labelled “lat. 23°”. Goyder (2016b) drew attention to problems localising Chapman & Baines’ collections particularly those labelled lat. 23° – if taken literally, this would imply they were collected near the Namibian coast E of Walvis Bay, but as this is desert and therefore unsuitable habitat for most of the species bearing these labels, it is much more likely that, given the well-documented route of the expedition, they originated in northern Botswana. Robyns (1928: 329) effectively lectotypified the name by citing the unlocalised “T.Baines” collection as the type.

## Supplementary Material

XML Treatment for Asclepias minutissima

XML Treatment for Cochlospermum adjanyae

XML Treatment for Endostemon palustris

XML Treatment for Baphia arenicola

XML Treatment for Vangueria fulgida

## Footnotes

Nomenclatural note on epithets commemorating Ilse von Nolde.

## ﻿Additional information

### Author ORCIDs

David J. Goyder https://orcid.org/0000-0002-3449-7313

Manfred Finckh https://orcid.org/0000-0003-2186-0854

Amândio Gomes https://orcid.org/0000-0002-7812-5490

Francisco Maiato P. Gonçalves https://orcid.org/0000-0002-8859-7491

Paulina Meller https://orcid.org/0000-0001-6711-4385

Alan J. Paton https://orcid.org/0000-0002-6052-6675

### Data availability

All of the data that support the findings of this study are available in the main text.
